# Life-Threatening Hemopericardium Associated with Rivaroxaban

**DOI:** 10.1155/2017/4691325

**Published:** 2017-04-05

**Authors:** Sijan Basnet, Niranjan Tachamo, Biswaraj Tharu, Rashmi Dhital, Sushil Ghimire, Dilli Ram Poudel

**Affiliations:** ^1^Department of Medicine, Reading Health System, West Reading, PA 19611, USA; ^2^Tribhuvan University, Maharajgunj Medical Campus, Kathmandu, Nepal; ^3^Universal College of Medical Sciences, Tribhuvan University, Bhairahawa, Nepal

## Abstract

Rivaroxaban is a novel oral anticoagulant used in the treatment of venous thromboembolism. The use of this medication has expanded over the recent years with resultant expansion of side effect profile. We present a case of a 56-year-old female who presented with shortness of breath and chest pain 12 weeks after starting rivaroxaban for left upper extremity deep vein thrombosis. She was later diagnosed with spontaneous hemopericardium requiring pericardial fluid drainage. Rarer side effects like this will be encountered more often with the increasing use of novel oral anticoagulants. The clinicians should be cognizant of this entity among patients with shortness of breath and chest pain for timely diagnosis and intervention.

## 1. Introduction

Rivaroxaban is a US Food and Drug Administration (FDA) approved novel oral anticoagulant for treatment of venous thromboembolism and for stroke prophylaxis in non-valvular atrial fibrillation. The use of rivaroxaban has been in the rising trend because of its ease of use as it does not require periodic monitoring. This increasing use has led to a rise in the observed side effects. Many bleeding side effects such as intracranial, gastrointestinal, or retroperitoneal bleeding have been associated with rivaroxaban [1].

Spontaneous hemopericardium with cardiac tamponade due to rivaroxaban, however, is a rare entity and only 4 cases have been reported so far [1–4]. Here we report a case of a 56-year-old female who presented with spontaneous hemopericardium secondary to rivaroxaban use.

## 2. Case

A 56-year-old female with a history of pulmonary embolism, myotonic dystrophy, and complete heart block on pacemaker presented to the emergency department (ED) with sudden onset shortness of breath and substernal chest pain radiating to the back. There was no history of fever, chills, cough, sore throat, or chest trauma. Her history was negative for any gastrointestinal, musculoskeletal, or neurological symptoms. She was taking rivaroxaban 20 mg once daily for deep vein thrombosis of the left axillary vein diagnosed 7 weeks ago. There was no prior history of tuberculosis, chest irradiation, or chemotherapy. She had a history of pulmonary embolism in 2007 for which she was on coumadin for a year. She had a biventricular pacemaker placed in 2008 for complete heart block induced nonischemic cardiomyopathy. Complete heart block was diagnosed at the same time and was believed to be secondary to myotonic dystrophy. The pacemaker device reached elective replacement indicator and was replaced with a Medtronic Protecta D314TRG device in 3/2012. She was on Tylenol 500 mg as needed for back pain, levocetirizine 5 mg twice daily for seasonal allergies, and rivaroxaban and zolpidem 5 mg nightly as needed. The above-listed medications do not have p-glycoprotein altering activity or CYP3A4/5 or CYP2J2 inhibiting activity [2, 5]. The past surgical history and family history were unremarkable.

**Figure 1:**
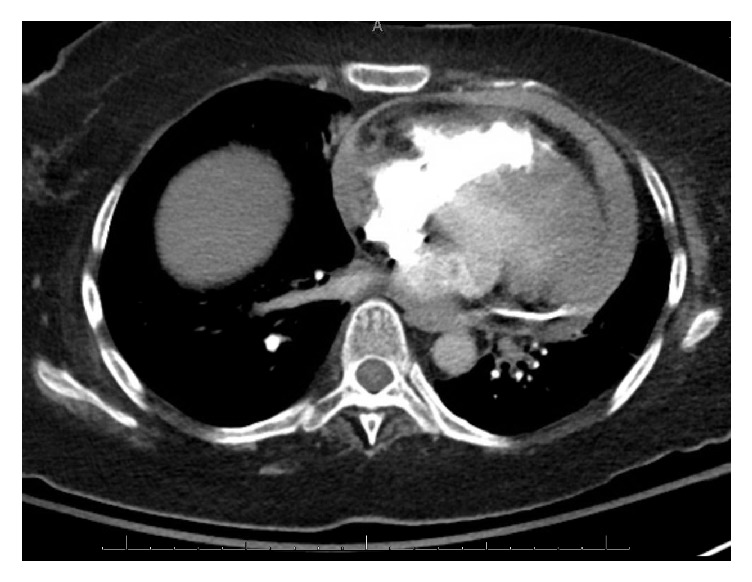
CT scan transverse view with hyperdense pericardial effusion.

**Figure 2:**
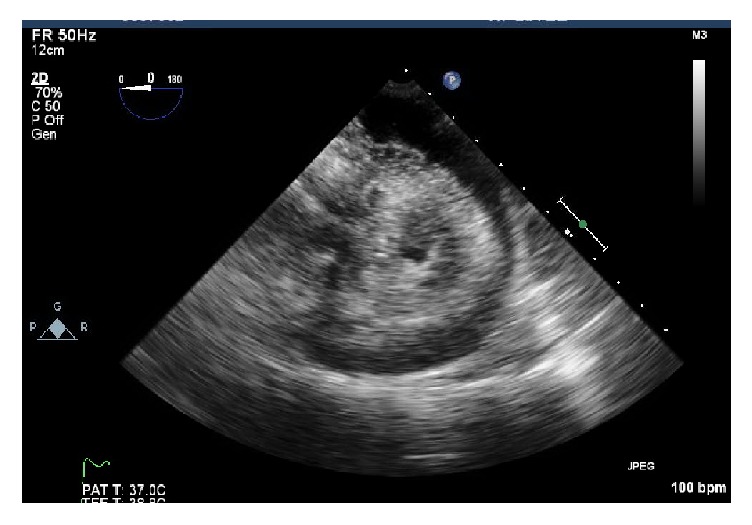
Echocardiography with pericardial effusion.

In the ED, her blood pressure was 78/48 mm Hg, pulse 106/minute, temperature 36.5°F, and respiratory rate 18/min with saturation of 87%. Her height and weight were 1.7 m and 180 lbs, respectively. Cardiac examination was normal. Chest examination was significant for bibasilar rales. Electrocardiogram revealed ventricular paced rhythm. There was no associated electrical alternans. Lab tests revealed negative troponin, INR of 1.3, and PTT of 28 s. Complete blood count and basic metabolic profile were unremarkable. Computed tomography (CT) on chest showed no pulmonary embolism but revealed a large hyperdense pericardial effusion (Figure 1). Echocardiogram showed moderate-sized circumferential effusion with a swinging heart consistent with pericardial effusion (prior echo 4 years ago showed no pericardial effusion) (Figure 2). Inferior vena cava was dilated with no respiratory collapse and right ventricle outflow tract collapsed in diastole. With the diagnosis of cardiac tamponade, emergency subxiphoid pericardial window was performed and a total of 300 mL of bloody pericardial fluid was drained. Hemostasis was achieved and was satisfactory. Transesophageal echocardiogram confirmed emptying of the collection as well as improved cardiac function. There was immediate improvement in her hemodynamic status. Postprocedure images showed complete resolution of the effusion. Further etiologic workup was unrevealing. Pericardial fluid culture was negative with normal cytology. Pericardial tissue biopsy was negative for malignancy. Thyroid and liver function tests were normal. ANA, rheumatoid factor,* anti-saccharomyces cerevisae* antibody, anti-mitochondrial antibody, C3, C4, and anti-liver-kidney-muscle antibody were negative making the autoimmune cause unlikely. Myotonic dystrophy was considered to be an unlikely cause of her hemopericardium as pericardial involvement has not been reported with it [6]. In addition, her previous echo was negative for pericardial effusion. There was no temporal relation to the axillary vein DVT and the pacemaker lead implantation as CT scan done in 2013 and echocardiogram done in 2015 mentioned a normal pericardium with appropriately implanted leads. Intraoperatively, there was no mention of pacemaker lead induced pericardial perforation. Further hospital course, unfortunately, was complicated by healthcare associated pneumonia with septic shock and multiorgan failure. The autopsy was not done.

## 3. Discussion

Rivaroxaban inhibits factor Xa and prothrombinase. This increases the propensity for bleeding in rivaroxaban users; however, the tendency for pericardial bleeding per se is unknown. Rivaroxaban has been approved for prophylaxis of stroke and systemic embolism in non-valvular atrial fibrillation, treatment and prophylaxis of DVT and PE, and prophylaxis of DVT following hip or knee replacement surgeries. The FDA approval of rivaroxaban for the above indications is supported by 3 trials: ROCKET AF trial, Bleeding Events in the Pooled Analysis of EINSTEIN DVT and EINSTEIN PE studies, and EINSTEIN extension clinical study. In these trials, a total of 16326 patients received rivaroxaban and the major bleeding reported was intracranial and/or gastrointestinal bleeding. There was a decreased risk of intracranial bleeding and an increased risk of gastrointestinal bleeding in patients on rivaroxaban compared to warfarin. Rivaroxaban had a statistically insignificant higher rate of major bleeding. In the Bleeding Events in the Pooled Analysis of EINSTEIN DVT and EINSTEIN PE studies, intracranial, retroperitoneal, intraocular, and intraarticular bleeding were observed, while in the EINSTEIN extension clinical study, gastrointestinal bleeding and menorrhagia were observed [5].

Hemopericardium, however, was not reported in any of these studies making it a rare form of lethal major bleeding side effect. Postmarketing experience has not identified it as an adverse effect either [5]. It might be due to the fact that this drug is relatively new and it is possible that increasing use of this drug might have led to the occurrence and detection of rarer adverse effect like spontaneous hemopericardium which were not detected in the clinical trials and postmarketing experience. Spontaneous hemopericardium in our patient was attributed to rivaroxaban after a thorough workup to exclude all the major etiologies [2–4, 6]. Spontaneous hemopericardium has also been associated with other novel oral anticoagulants (NOACs) like dabigatran and apixaban [7–10]. Hemopericardium has been reported with vitamin K antagonists but no studies comparing NOACs to these medications have been done yet [11].

Evaluation for pericardial effusion with an echocardiogram in a patient on rivaroxaban who presents with unexplained shortness of breath seems to be a reasonable recommendation we can make. As echo has a very good sensitivity and can detect as low as 20 mL of pericardial fluid, it can be considered a reliable diagnostic modality in these patients [12]. The biggest concern with the widespread use of rivaroxaban is that it does not have an approved reversal agent or lab test to predict its anticoagulant effect [6]. It is reasonable to stop the responsible anticoagulant, assess the risks and benefits, and resume another anticoagulant if needed [8].

## 4. Conclusion

Rivaroxaban, like other anticoagulants, is associated with increased bleeding risk. Spontaneous hemopericardium, including cardiac tamponade due to novel oral anticoagulants, is a rare side effect and only few cases have been reported so far. Clinicians should be aware of this little known but life-threatening adverse effect of rivaroxaban, so that they can identify these patients early and institute appropriate interventions which can be lifesaving. Whether periodic echocardiogram should be done in these patients to identify early pericardial effusion needs further research.
